# Spatial Concentration of Health Industry under COVID-19

**DOI:** 10.3390/ijerph20054444

**Published:** 2023-03-02

**Authors:** An-Ting Cheng

**Affiliations:** Department of Land Economics, National Chengchi University, Taipei 11605, Taiwan; acheng@nccu.edu.tw

**Keywords:** health-related manufacturing industry, spatial distribution, industry cluster, COVID-19 pandemic, industrial survival, life cycle, agglomeration, Taiwan, knowledge intensive industry

## Abstract

The COVID-19 pandemic has impacted the industry immensely and, in some cases, irreversibly. This research pioneers in studying how the pandemic have influenced the survival and spatial distribution of the health-related manufacturing industry (HRMI) in Taiwan. Eight categories of HRMI are examined, with their change in survival performances and spatial concentration between 2018 and 2020. Average Nearest Neighbour and Local Indicators of Spatial Association are conducted, to visualise the distribution of industrial clusters. We found the pandemic did not shock the HRMI in Taiwan, but actually induced its growth and spatial concentration to a certain extent. Additionally, due to it being a knowledge-intensive industry, the HRMI mainly concentrate in metropolitan areas with which universities and science parks may have largely supported. However, the spatial concentration and cluster scope growth do not necessarily accompany the improvement of spatial survival, which may be resulted from the different life cycle stages an industry category is in. This research fills in the gap of medical studies with literatures and data from the field of spatial studies. It provides interdisciplinary insights under the condition of pandemic.

## 1. Introduction

For the past few years, industries in all walks of life around the globe have experienced shocks from the COVID-19 pandemic and require in-depth research to understand the cause-and-effects, details, and consequences, to be able to accumulate experiences for the future. The purpose of this research is to provide such experience for the medical industry, by studying how COVID-19 impacted Taiwan’s health-related manufacturing industry (HRMI), from the perspective of spatial distribution. By analysing data during 2018–2020, we examine the change in spatial concentration and survival performances of the HRMI, to conclude in what ways has COVID-19 impacted Taiwan’s health industry. It fills the academic gap of medical studies, by (1) approaching it from spatial analysis and knowledge; (2) analysing medical industries under the emergent situation of pandemic; and (3) discovering unique patterns generated by a knowledge-intensive industry in which the medical industry belongs.

This research is organised into the following sections:

- Literature review—We first look into theories surrounding spatial concentration and industrial survival. As there exist both supportive and challenging arguments regarding whether agglomeration leads to greater chances of survival, we search for evidence whether the life cycle of industry can be another factor affecting survival performance. We notice that regardless of arguments, past literatures have only looked into the performance of firms as research objects, therefore we select production sites as target of study, to simplify the factors. As the medical industry, especially during the time of pandemic, requires substantial knowledge to support the industry, we present literatures regarding knowledge-intensive industries.
- Research concept—This section explains the details of our research: what we are going to evaluate, based on what indicators, and what to explore. To avoid confusion, we clarify the use of “agglomeration” and “clusters” that come with different nuances.
- Case study of Taiwan’s health manufacturing industry—This section provides a practical insight of the current trends of Taiwan’s HRMI, including market growth and governmental policies. To understand the spatial distribution of HRMI, the section provides the map of current major medical universities and science parks, to explain their connection with the health industry, by linking with the literature review regarding knowledge-intensive industries.
- Research method—Explains the concept of Average Nearest Neighbour, Local Moran’s I, as well as COType.
- Research data—Providing the source of our data, and Section 4.2 that defines the HRMI categories.
- Research findings—Tables of statistics that include survival performance, z-score of spatial distribution, LISA analysis, visualised map of spatial concentration, and spatial operation preference.
- Discussion—Illustrates noteworthy issues from the findings. We discover that the pandemic has not created serious impact toward the HRMI, but the positive correlation between spatial concentration and survival performance was not found. Regarding the distribution of the HRMI, it is found that they predominantly match the locations of medical institutes and science parks, and thereby we conclude that especially during the urgent circumstance of pandemic, these organisations may have assisted the HRMI, maintaining its performance.
- Conclusions.

## 2. Literature Review

### 2.1. Spatial Concentration and Industrial Survival

Spatial concentration, “agglomeration” or “clusters” refer to the highly-dense state of a particular system within a given space. Agglomeration of firms in a common industry has the benefit to realize inter-firm spill-overs in sharing technology, inputs and customers [1]. There exist competing opinions, whether spatial concentrations are always beneficial to industrial survival. In general, agglomeration of economic activities could lead to various externalities, and further accelerates the development of industrial clusters. Porter [2] considers industrial agglomeration to be promotive for regional competitiveness, and regional economic growth demonstrates positive effects on industrial development. Some scholars believe that clusters are advantageous to industrial development, as they provide better access toward resources and knowledge exchange networks that facilitate technology spill-overs [3]; the improvement of labour productivity [4,5]; and lower production costs [6]. When economies are shocked, industrial agglomerations and clusters are more advantageous to resilience [1,7].

According to Cainelli et al. [8], economies with agglomeration significantly attenuate firm mortality in industries. Staber [9], on the other hand, indicated that clusters of firms in the same industry would increase business failure rates, but firms operating in complementary industries in diversified clusters would reduce failure rates. Behrens et al. [10] state that they found no evidence for industries in clusters to be more resilient than those outside, although the result might match the claim of Staber [9], as the clusters belong to a same industry, and also that there are other factors such as international policy frameworks or trade protections that affect local productions. From above literature, we may summarise that although there are more literatures and evidence agreeing that agglomeration leads to longer industrial survival compared to those holding opposite views, it is not appropriate to assume that clusters are always beneficial to industries, and there are more factors to be looked into.

### 2.2. Survival Performance at Each Stage of Industry Life Cycle

Notably, some researchers remind that the performance of firms varies with the evolution of industry and the stage of industrial life cycles [11,12], so a question arises about whether the firm mortality or failure rates are affected by the life cycle an industry is in. The connotation to be discussed in this study is the life cycle of an industry, rather than industrial clusters/agglomerations. Industry life cycles usually go through the stages of “introduction”, “growth”, “maturity” and “decline”. Williamson [13] found out that as the early stages involve the design and manufacturing of new products, a high degree of uncertainty characterizes these formative stages. By contrast, in the mature and declining stages, the industry evolves towards standardisation, and markets tend to grow at a more stable rate. According to Agarwal [14], probability of survival significantly differs across evolutionary stages. Early entrants enjoy a higher probability of survival, although the probability does not monotonically decrease with age. The size of the firm in the period prior to exit has a negative impact on hazard rate. Large sized firms have a lower failure rate across all ages, possibly due to superior endowments. Small firms that grow before they exit survive on average three times longer than firms who do not exhibit any growth. Esteve-Pérez et al. [15] found that in the early stage of the life cycle, firm age is negatively correlated with hazard rates while firm productivity is not. Firm productivity is associated with lower hazard in the maturity stage, when competition is primarily efficiency-driven. Wang et al. [16] suggest that agglomeration attracts more new entry in the growth stage, whereas it contributes to firm survival in the mature stage, but there is little empirical evidence that proves the surviving firms are qualitatively different at different stages of the evolution [17].

Regarding the research mentioned above, whether agreeing that spatial concentrations can enhance the survival chance or not, their research objects all focus on the firms. However, in reality, even if a firm fails after shock, the production could continue in the same location by another operator. In other words, from the spatial point of view, the industry is considered resilient since its economic activities are not interrupted after shock. Therefore, focusing on the economic performance of production sites may be more accurate in evaluating industrial resilience. In this research, the performances of production sites rather than individual firms are evaluated.

### 2.3. Knowledge-Intensive Industry and Pandemic Control

The above literatures describe the spatial and life cycle theories for industries in general. However, we further investigate whether different types of industry generate unique patterns of spatial distribution and its growth under certain conditions. Literature has suggested that knowledge-intensive industries might generate unique spatial and survival patterns, as they rely on knowledge spill-over, and since the medical industry belongs to a highly knowledge-intense industry, this research considers being knowledge-intensive a factor influencing the survival of the HRMI. Meanwhile, it also makes a comparison with non-knowledge-intensive industries, to discusses the differences in the spatial concentration between knowledge-intensive industries and non-knowledge-intensive industries.

Knowledge-intensive industries and their knowledge-driven economies generate positive effects on the regional level and have increasingly high importance in developmental states [18]. Bolter and Robey [19] state that knowledge-based industries are those who benefit the most from agglomeration, as sharing ideas are central to the production process. Duschl et al. [20] investigated the industrial clustering and firm growth under an industry-specific scope, and after analysing 23 different industries they found that proximate scientific activities generate different impacts on industries, depending on the type and age of industry. Ženka et al. [21] suggested that urban cores are highly attractive for creative industries and technology-based industries.

The theory from Porter [2] highlighted four key reasons for the success of industrial districts: “factor conditions” which concerns cost and the quality of labour; “demand conditions” which relies on specific concentration of consumers; “related and supporting industries”; and “firm strategy structure and rivalry”. However, this theory only looked into the impacts from other profit-oriented industries toward a certain industry, while in reality, other forms of influential players, such as governments or academic institutions might also play a big role toward the wellbeing of an industry. Breznitz and Anderson [22] have found that universities play a key role in knowledge intensive industries, as the academic institutes contribute the human capital, and in industries such as biotechnology create the technological innovations that can be commercialised by local firms. Thus, we can say, the presence of neighbourhood universities and academic institutions might be a decisive factor that differentiates knowledge-intensive industries from non-knowledge-intensive industries.

The phenomenon of knowledge spill-over is unique and essential for knowledge-intensive industries, as knowledge-intensive suppliers apply or combine existing knowledge elements, to create products based on new knowledges [18]. To realise this, the source of such knowledge, skill, experiences should locate in the vicinity with minimum distance, in order to be delivered with minimum cost and time. This generates a unique spatial pattern for knowledge-intensive industries.

University–industry collaborations is a typical landscape for medical industries. Baba et al. [23] state that the analytical knowledge base (which requires creation of new scientific knowledge, as in the case of biotechnology and pharmaceuticals) is typical of industrial settings where scientific knowledge is fundamental for innovation. The health industry is strongly dependent upon academic institutions, as university-to-industry is the dominant direction of knowledge flows. COVID-19 is a situation of emergency that poses high risks and uncertainties for industries, which requires a high degree of flexibility and resilience to manage the risks, for achieving a controllable production rate [24]. In our study, we discover that health industries, especially those concerning digital technologies, can be greatly impacted from proximate scientific activities, especially in the era of pandemic. To minimise the health-related hazards of the general public, constantly updated information of transmission and pioneering medical knowledge is required, such as generation of new variants of the virus, and medical treatments against different symptoms. Bartnicka et al. [25] informed the importance of staff quality under emergency circumstances such as pandemic. As industries have to swiftly adapt to survive, the training of human resources requires a high standard of safety and knowledge, thus it is reasonable for R&D efforts to be geographically concentrated, to generate radical innovation [18]. As researchers of COVID-19 are highly knowledge-intense and require frequent updates, hospitals and medical institutes serve as the forefront of health information management, and the HRMI would need to be located in the vicinity of these constructions to obtain the knowledge needed in order to benefit the business and support the industries.

## 3. Research Concept

This study evaluates the survival performance of Taiwan’s HRMI, based on the longevity of production sites, to explore spatial concentration in relation to industrial survival, and thereby look into whether spatial patterns impact the long-term survival performance of the industry. As the boundaries of industrial clusters are not easy to grasp, we will define the clustered and non-clustered areas by a spatial analysis tool, and thereby compare the long-term survival performance of the two. The “industrial space survival” this study proposes differentiates from common corporate survival. The former focuses on the production in a space, not limited to the survival time of a single enterprise, but whether an industry continues to produce in terms of spatial location; while the latter focuses on the death of a single enterprise, and mostly aims to improve the survival time to explore possible influencing factors, such as production location, life cycle, market strategy, etc. For example, if manufacturer B in space A stopped production due to a certain impact, but manufacturer C continued to enter space A to continue production, it can be said that the industry in space A continued to survive, but the company failed to survive. In other words, the survival of industrial space can reduce the influencing factors of enterprise survival to a certain extent and can explore the performance of industrial survival in a more macroscopic manner.

On the other hand, we explore the spatial survival and spatial distribution changes of the health industry during the outbreak of COVID-19, including the concentration of the overall spatial distribution, the distribution of concentrated areas and the spatial preference of industrial operations.

### Terminology Clarification

To clarify the use of terminology, both “agglomeration” and “cluster” refer to the state of industrial concentration. In most literature regarding industrial regions, agglomeration occurs earlier than cluster. Agglomeration usually describes similar industries gathered together to achieve better economic benefits, but clusters further develop advantages such as knowledge spill-over. In other words, a cluster must be an industrial agglomeration, but agglomeration does not necessarily achieve the effect of a cluster. Secondly, as our research uses spatial analysis, it may not be possible to know whether the concentration of the industry belongs to agglomeration or cluster, because it is not possible to verify whether there is any effect of knowledge spill-over among enterprises. From this point of view, “agglomeration” can be a more suitable term. However, “cluster” rather than “agglomeration” is the term used in ArcGIS analysis (for example, the LISA analysis we made is called “cluster and outlier analysis” in the system), so in related discussions about research analysis, it may not be appropriate to change “cluster” to “agglomeration”. Based on the above three points, “cluster” here is regarded as a popular term, for describing industrial aggregation, and when explaining the spatial characteristics of aggregation or referring to specific literatures, “agglomeration” is used.

## 4. Case Study: Health Manufacturing Industry in Taiwan

### 4.1. Current Trends

**Chart 1:**
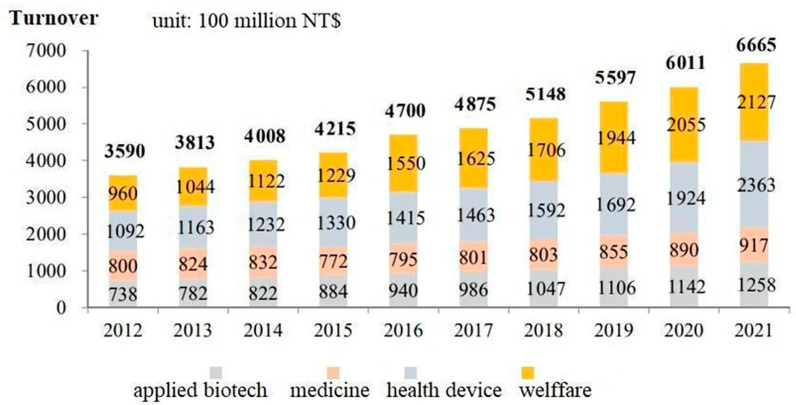
Growth trend of Taiwan’s biotechnology turnover Source: Ministry of Economic Affairs, Medical and Pharmaceutical Industry Technology and Development Center, Industrial Technology Research Institute, 2022.

Statistics show the COVID-19 pandemic initiated in 2020 has not threatened the health market in Taiwan but further stimulated it. Statistics from the Ministry of Economic Affairs shows that in Taiwan, the turnover of health devices has grown from TWD 192.4 billion in 2020 to TWD 236.3 billion in 2021, with a growth of 22.8% [26]. As shown in Chart 1, the growth rate since the start of the pandemic does not differ much from the pre-pandemic era.

The literature from Section 1 has emphasised the importance of academic units in the vicinity of industries, to assist their performance, but besides medical universities, science parks can also be an important helping force supporting and promoting the HRMI. For example, in 2020, the Southern Taiwan Science Park (consisting of Tainan Science Park and Kaohsiung Science Park) assisted 17 biotech firms to hold an exhibition [27]. According to recent reports, the science park is devoted in collaboration with hospitals and medical institutes, to promote the local production of medical devices. From a viewpoint of governance, as most science parks are owned and managed by the government sector, we might infer that there is a driving force to push the national industries forward, promoting their performances nation-wide and even overseas.

**Figure 1:**
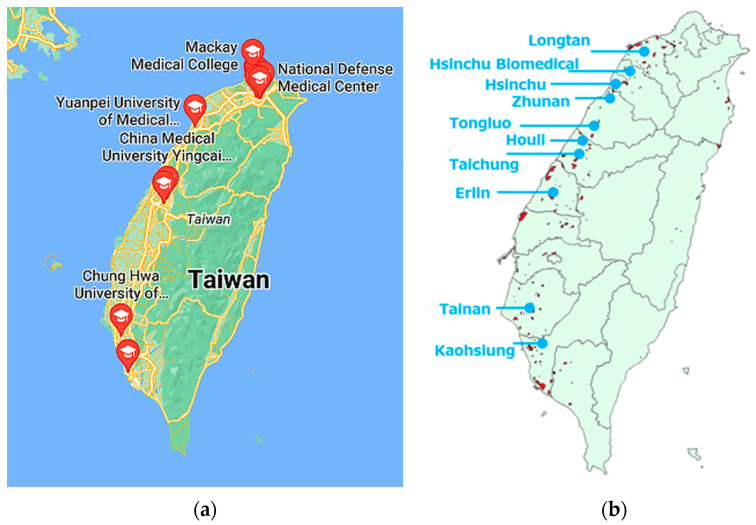
Distribution of (**a**) major medical institutes and (**b**) main science parks in Taiwan. Source (**a**) Google maps Source (**b**) Translated from site https://portal.stpi.narl.org.tw/index?p=article&id=4b1141427395c699017395c756b31f82 (accessed on 19 January 2023).

**Figure 4:**
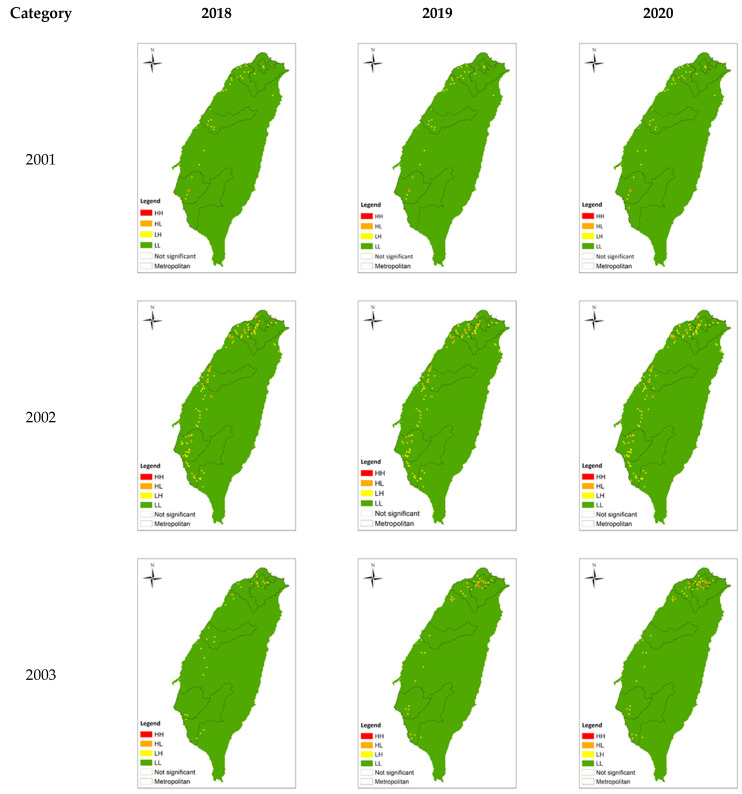

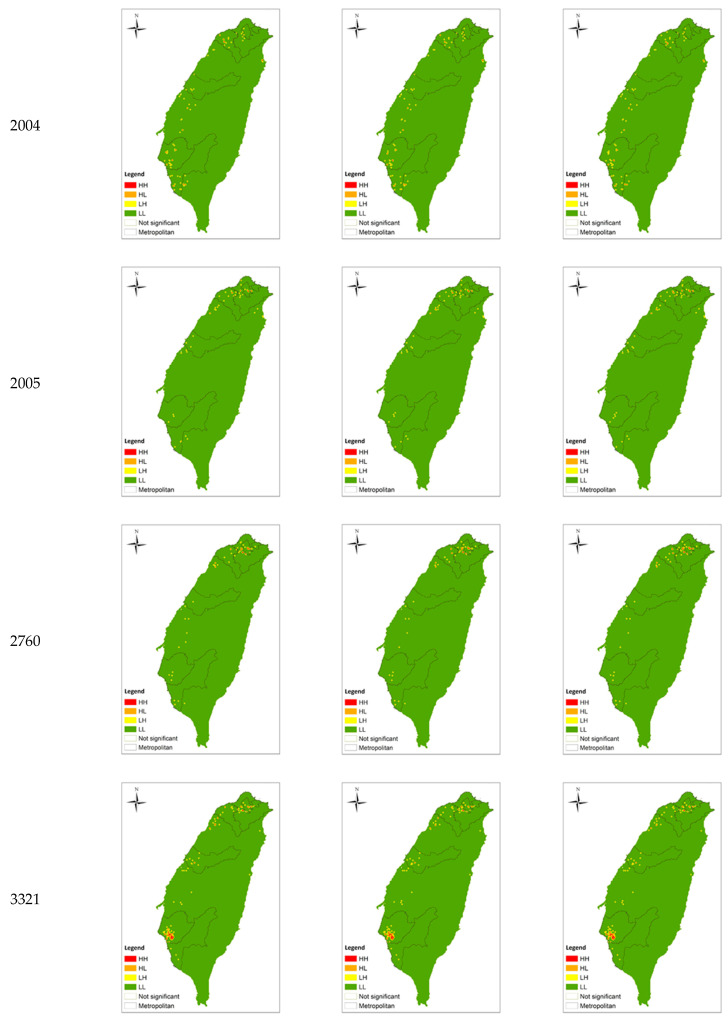

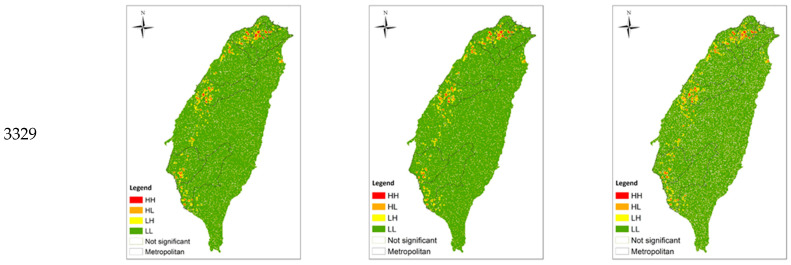
Distribution of industrial spatial concentration.

Below is the distribution of (a) major medical institutes and (b) main science parks in Taiwan, as shown in Figure 1. Both of them are located along the Western coast, with the medical schools concentrating on metropolitans such as Taipei, Taichung and Kaohsiung. The distribution generally matches with the LISA analysis shown in Figure 4 which visualises the distribution of the HRMI in Taiwan from year 2018 to 2020.

To provide an overview of health policies for the future, the Taiwanese government has placed digital health a priority for the future health industry. In 2021, “Act for the Development of Biotech and Pharmaceutical Industry” supports the development of several new fields of Medicare, including new drugs, high-risk medical equipment, regenerative medicine, precision medicine, digital medicine, contract development and manufacturing companies (CDMO), and innovative technology platforms [28]. In 2022, the Ministry of Science and Technology launched the “Smart Medical Industry-University Alliance Program”. It integrates multiple different sectors and resources such as the Ministry of Economic Affairs and the Ministry of Health and Welfare, the National Health Insurance Administration, and science parks, to encourage collaboration, effectively implementing smart health [29]. Digital health improves the accuracy in diagnosis and disease treatment and enhances the delivery of health care. It includes mobile health, health information technology, artificial intelligence (AI), wearable devices, telehealth and personalised medicine [30]. AI health can assist doctors in handling tedious, repetitive works. For example, chatbots and wearable technologies can record the consultation process, and automatically transfer the data to electronic health records [31].

According to the data from the Industrial Technology Research Institute, in 2021, Taiwan’s digital medical turnover was TWD 45.56 billion. Compared with TWD 41.2 billion in 2020, the annual growth rate was 10.6% [26]. The institute divides the digital medical industry into five core aspects of “digital prevention”, “digital diagnosis”, “digital treatment”, “remote health” and “medical information system” as the key direction of industrial development. Operating upon virtual platforms, digital health fulfils the needs due to the growing pace of ageing and increased travel opportunities. Liang and Lin [32] found that for rural areas, remote digital health systems provide the medical resources needed for long term and elderly care. For cities, it relieves hospital overcrowding by balancing patient counts, while for those travelling overseas, remote health avoids expensive charges from local hospitals due to the lack of local health insurance. “My Health Bank” is a digitised health monitoring system promoted by the Ministry of Health and Welfare (MOHW) in Taiwan. The database records one’s history of healthcare, diagnosis, medicine and vaccination, payments etc [33]. The technology utilises cloud computing and software development kits, connecting multiple apps from third parties such as hospitals and pharmacies.

### 4.2. Research Data

The data of the status and operation sites of the Taiwanese health manufacturing industry from 2018 to 2020 are provided by the Industrial Development Bureau of the Ministry of Economic Affairs (Taiwan). For the purpose of this research, we have included data from the following eight industry categories: 2001, 2002, 2003, 2004, 2005, 2760, 3321, and 3329, as targets of study, presented in Table 1. It includes both digital and non-digital health products, but all of them require a high level of medical and biotechnological knowledge to be manufactured, thus in this research the below categories are all considered as knowledge-intensive industries.

## 5. Research Method

This research uses the quantitative methods of Average Nearest Neighbour and Local Moran’s I to analyse spatial distribution of the eight categories of the HRMI presented in Table 1. After that, we compare the spatial distribution data with survival performance data of the eight categories, which is presented in Section 6.

### 5.1. Average Nearest Neighbour

**Figure 2:**
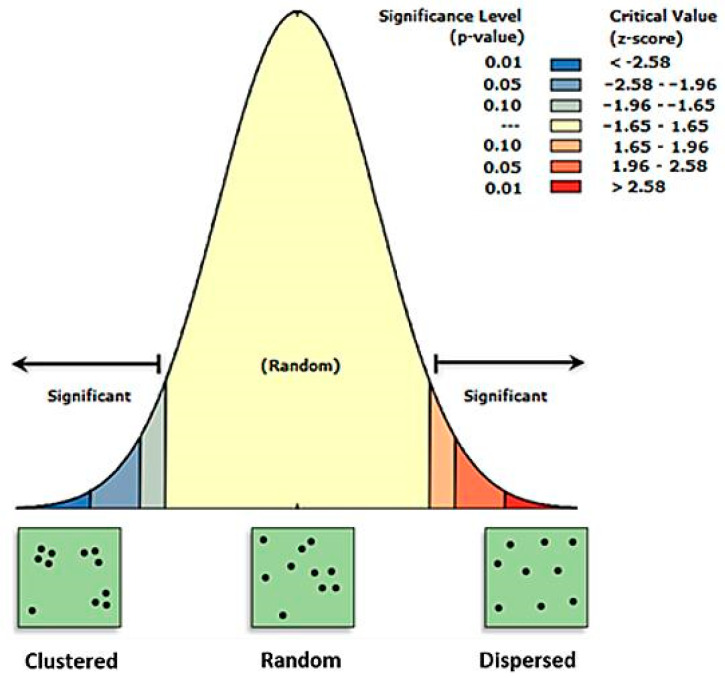
Average nearest neighbor analysis Source: Edited figure from Esri ArcGIS (https://desktop.arcgis.com/en/arcmap/10.3/tools/spatial-statistics-toolbox/average-nearest-neighbor.htm (accessed on 13 January 2023)).

Average Nearest Neighbour (ANN) measures the distance between centroids, namely between the centroids of a particular feature and the centroid of its nearest neighbour. These results are then averaged. If the average distance is less than that of a random distribution, it is considered clustered; if greater, then considered dispersed. The ANN ratio represents the observed average distance divided by the expected average distance. Below are the formulae of Z-score calculation:

(1) z=D¯0−D¯E0.26136n2A

(2) $${\bar{D}}_{0}=\frac{\sum_{i=1}^{n}\mathrm{di}}{n}$$

(3) $${\bar{D}}_{E}=\frac{0.5}{\bar{\mathrm{Jn}∕A}}$$

where ${\bar{D}}_{0}$ is the observed mean distance between each feature and its nearest neighbour, ${\bar{D}}_{E}\;$ is the expected mean distance for the features given in a random pattern. In the above equations, di equals to the distance between feature i and its nearest neighbouring feature. n corresponds to the total number of features, and A is the area of minimum enclosing rectangle around all features. If the z-score is less than −1.65, the pattern exhibits clustering; if the z-score is greater than 1.65, the trend is toward dispersion (as Figure 2).

### 5.2. Local Moran’s I

Spatial autocorrelation is the study of calculating the extent of spatial autocorrelation between a certain spatial unit and its surrounding units, upon a particular characteristic value through statistical methods, to analyse the spatial distribution of these spatial units. Among them, LISA (Local Indicators of Spatial Association) analysis proposed by Anselin [34] measures the extent of influence of spatial units on the spatial autocorrelation of the entire research area, to calculate the range of the spatial hot spot, as those with a greater degree of influence are often the “outliers” in the region, and most of these exceptional “outlier” points belong to the spatial phenomena of agglomerated points. LISA can be calculated by Local Moran’s I, which the statistic of spatial association can be presented as:

(4) $$I_{i}=\frac{x_{i}-\bar{X}}{S_{i}^{2}}\overset{n}{\underset{j=1,j\neq i}{\sum }}w_{i,j}\left(x_{j}-\bar{X}\right)$$

where $w_{i,j}$ is the spatial weight between feature i and j; $x_{i}$ is an attribute for i, $\bar{X}$ representing the mean of the attribute, and:

(5) $$S_{i}^{2}=\frac{\sum_{j=1,j\neq i}^{n}{\left(x_{j}-\bar{X}\right)}^{2}}{n-1}$$

where n is the total number of features.

**Figure 3:**
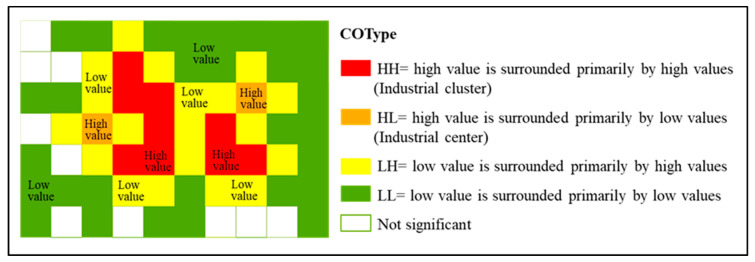
Distribution analysis of Industrial spatial concentration (Local Moran’s I).

When the test reaches a significant level and there is significant positive regional spatial autocorrelation, that is, a spatial unit is surrounded by those with similar attribute values, it is called a “cluster”. The cluster/outlier type (COType) distinguishes four spatial types that describe the density of a spatial unit in relation to the density of its adjacent units: HH, LL, HL and LH. When the observed value of the spatial unit and the adjacent spatial unit are both high, the COType is expressed as High–High (HH), which is regarded as the spatial agglomeration unit of a specific industry in the year. When the observed value of the spatial unit and the adjacent spatial unit is both low, the COType is expressed as Low–Low (LL), which is regarded as a discrete unit in the space of a specific industry in that year. In addition, a space unit surrounded by space units with different attribute values is called an “outlier”. When the observation value of the space unit itself is high, but the adjacent space is low, the COType is expressed as High–Low (HL), which is regarded as the spatial concentration unit of a specific industry in the year. When the observation value of the space unit itself is low, but the adjacent space is high, the COType is expressed as Low–High (LH), which is regarded as the diffusion unit of the specific industry in the year (as shown in Figure 3 below).

## 6. Research Findings

In this section, the survival performance (Table 2), spatial concentration growth rate (Table 3), spatial agglomeration units (Table 4 and Figure 4), and spatial operation preference (Table 5) of the eight HRMI categories are presented, to draw comparison and look into the correlations.

### 6.1. Industrial Survival in Space

According to the 2020 statistics shown in Table 2, the production site of category 3329 had the highest number, exceeding 800 sites. The upcoming categories were 3321 and 2002, each having around 200~300 production sites, followed by category 2004 and 2760 with around 100 production sites for each. The industries with the least production sites are categories 2001, 2003, and 2005, each numbering around 50. Almost all categories reached more than 80% of spatial survival rate in 2020. The highest rate was category 3321 and 2003, exceeding 90%; followed by categories 2002, 2004, 2005, and 2760, reaching over 85%. The spatial survival rate of category 2001 was about 80%, while that of category 3329 was only about 75%.

Looking at the development and changes of various categories, the number of production sites from 2018 to 2019 showed a growing trend, with category 3329 growing the most, followed by categories 3321 and 2002. However, after entering 2020, the growth trend of the HRMI has slowed down, and some categories have experienced negative growth, such as categories 2001, 2003, 2760, and 3321. On the other hand, the spatial survival rate of various categories maintained a growth trend in 2020, among which category 2003 had the highest growth rate, with a growth rate of more than 35%, followed by category 2760 and 2001, with a growth rate of about 16–20%. Categories 3321, 2005, 2004, and 2002 had a growth rate of about 5–10%, and that with the smallest growth rate was category 3329, growing around 1.09%. Overall, the spatial survival rate in 2020 improved significantly compared to 2019.

### 6.2. Spatial Analysis

#### 6.2.1. Global Distribution of Industrial Spaces

When calculating the overall (global) spatial distribution of the HRMI by Average Nearest Neighbour (ANN) analysis, if the z-score is negative and lower, the spatial distribution tends to be more agglomerated, and it is a dispersed distribution if otherwise. If the z-score is between −1.65 and 1.65, it is randomly distributed. The data in Table 3 shows that the overall spatial distribution of each category in 2020 is characterised by concentration. The category with the highest concentration is category 3329, followed by categories 3321, 2002, 2004, 2760, 2005, 2001, and 2003.

Looking at the changes in the spatial concentration of categories from 2018 to 2020, except for the decrease in category 2004 and the roughly equal concentration of categories 2005 and 3321, the overall spatial distribution of other categories tended toward concentration, especially categories 2001, 2003, and 3329 that had the largest growth rate. Comparing the changes of spatial concentration from 2018 to 2019, except for categories 2004, 2005, and 3321, which were roughly flat or declining, the spatial concentration of other categories increased significantly. From 2019 to 2020, the change in spatial concentration was not obvious compared with the previous year. Except for category 3329, most of them showed a trend of flat or declining concentration.

#### 6.2.2. Distribution of Industrial Spatial Concentration

To further understand the location distribution of spatial concentration of health-related industries, this study explores the location and scope of the spatial concentration distribution of industries through Local Moran’s I.

According to Local Moran’s I analysis (Table 4 and Figure 4), the following describes the spatial agglomeration and concentration distribution of each industry category and the changing trend from 2018 to 2020:

- Category 2001: Manufacture of Raw Material Medicines

The number of spatial agglomeration units (HH) of category 2001 remained unchanged, and the location was fixed in the southern metropolitan area and one non-metropolitan area. The number of spatial concentration units (HL) had a growing trend. In addition to the fixed distribution in various metropolitan areas and some non-metropolitan areas, there was an increase of one in the non-metropolitan area adjacent to the northern metropolitan area in 2019, and one disappeared in the central metropolitan area in 2020. In the southern metropolitan area and the non-metropolitan area between the central and southern metropolitan areas, the newly added area had a gradual trend towards the south as a whole.

- Category 2002: Manufacture of Drugs and Medicines

The number of HH of category 2002 decreased for one location in the southern metropolitan area, which was reduced to a spatially concentrated unit, while the rest of the locations were fixedly distributed in various metropolitan areas and some non-metropolitan areas. The number of HL tended to increase year by year. In addition to the fixed distribution in the metropolitan areas, the newly added areas were mainly in the northern metropolitan areas and adjacent non-metropolitan areas, and the disappearing areas were mainly in the southern metropolitan areas.

- Category 2003: Manufacture of Medicinal Biological Products

The number of HH of category 2003 remained unchanged, and the location was also fixed in the northern metropolitan area and one non-metropolitan area. The number of HL had a slight growth trend, including one addition in the central metropolitan area and one location shift in the northern metropolitan area.

- Category 2004: Manufacture of Chinese Medicines

The number of HH of category 2004 decreased year by year. Except for the reduction and location shift in the northern metropolitan area and the southern metropolitan area, it was roughly fixed in various metropolitan areas and some non-metropolitan areas. The number of HL shows a growing trend. It also increased, decreased, and shifted in the northern metropolitan area and southern metropolitan area, and was roughly fixed in all metropolitan areas and some non-metropolitan areas.

- Category 2005: Manufacture of Medicinal Chemical Products

The number of HH of category 2005 increased year by year, and the distribution and newly added areas were located in the northern metropolitan area and the adjacent non-metropolitan area. The number of HL was also a growing trend, and its fixed distribution and changing range extended to all metropolitan areas and non-metropolitan areas on the periphery of metropolitan areas.

- Category 2760: Manufacture of Irradiation and Electromedical Equipment

The number of HH of category 2760 increased year by year, and the distribution and newly added areas were located in the northern metropolitan area and the non-metropolitan area adjacent to the northern region. The number of HL tended to decrease and was fixedly distributed in various metropolitan areas and some non-metropolitan areas, while the scope of change was mainly in the northern metropolitan areas.

- Category 3321: Manufacture of Eyeglasses

The number of HH of category 3321 decreased year by year, and was fixedly distributed in various metropolitan areas and some non-metropolitan areas, while the scope of change was mainly in the northern metropolitan areas and southern metropolitan areas. The number of HL was increasing, and the fixed distribution and changing range involved all metropolitan areas and some non-metropolitan areas.

- Category 3329: Manufacture of Other Medical Instruments and Supplies

The number of HH of category 3329 increased year by year, and the fixed distribution and changing scope involved all metropolitan areas and some non-metropolitan areas. The number of HL also increased and was also fixed and changing in various metropolitan areas and some non-metropolitan areas.

Overall, the spatial agglomeration units of health-related industries were mainly metropolitan areas and their peripheral non-metropolitan areas. Except for category 2004 and category 3321, where the scope of industrial space (HH + HL) decreased, the rest of the categories showed an increasing trend. Looking at the two types of spatial units separately, the categories with increasing numbers of spatial agglomeration units (HH) include categories 2005, 2760, and 3329, and the rest were flat or decreasing; the number of spatial concentration units (HL) was all increasing, except for category 2760.

#### 6.2.3. Spatial Operation Preference

Judging from the quantity ratio of two types of spatial units, the spatial operation preference of the industry Pr = HHn (the number of HH spatial units)/HLn (the number of HL spatial units). When Pr >1, it means that the manufacturers tend to operate in agglomeration, and Pr < 1 indicates the manufacturers tend to operate independently. Overall, the number of spatial concentration units (HL) of categories 2001, 2002, 2003, 2004, 2005, and 2760 between year 2018 and 2020 is higher than the number of spatial cluster units (HH), showing that these categories prefer independent operation. Category 2760 is the only exception, with the Pr value gradually approaching 1, indicating the trend towards agglomerated operation for the future. On the other hand, the number of HL of categories 3321 and 3329 is lower than the number of HH, indicating that the categories prefer clustered operations, but the Pr value of category 3321 is gradually shrinking, suggesting that it may turn independent in future business trends.

## 7. Discussion

Comparing the number of production sites and the performance of spatial survival in different industries, it is found that industries with a large number of production sites do not necessarily have better spatial survival performance. The relation between spatial concentration and survival performance did not show a positive correlation, indicating that agglomeration does not always contribute to higher survival rates for industry, which reflects the findings from Behrens et al. (2020). However, real situations are often more complex, and other factors may exist that affect survival performances.

To look at some examples, category 3329 had the largest number of production sites, and both the industrial spatial concentration and the number of industrial agglomeration units were growing positively, but the survival rate of the category was the lowest among all industries. As Agarwal (1997) points out that the life stage an industry is in affects its survival performance, analysing from life cycle theories, this can indicate that the life cycle of category 3329 might be in a relatively early stage, thus the growth momentum was strong, but the survival status was unstable. 

On the other hand, for category 3321 and 2003, which had the highest spatial survival rate, although the number of production sites grew from 2018 to 2020, the spatial concentration and the number of agglomeration unit were flat or even declining, which may reflect that the life cycle of category 3321 and 2003 entered a relatively mature stage, thus the growth momentum was slowing down, and the survival status was stable. The above examples match the claim of Williamson (1975) that the growth of industries in the mature stage tend to be more stable than those in early stages. 

From another point of view, looking into the details of each industrial category, category 2003 stands for the manufacturing of medical biological products, such as biological drugs and vaccines. Among all, this particular category enjoyed the highest growth of spatial survival rate during 2019–2020, compared with 2018–2019, with the growth rate of over 35%. From the nature of this manufacturing category, we might infer that its outstanding growth can be attributed to the pandemic taking place in 2020, as the products of category 2003 are those more likely used for pandemic prevention and treatment, compared with other categories.

Based on the national data until 2020, we observe that the first stage of COVID-19 pandemic has not significantly impacted the spatial survival of the health industry in Taiwan in negative ways. However, due to the fact that different timelines exist for the outbreaks of each country, and the outbreak in Taiwan started in the first half of 2022, much later than the rest of the world, how the pandemic continues to impact the spatial distribution of the HRMI in Taiwan remains as an observation target for future researchers. For the time being, we may only conclude that the pandemic on a global scale has not negatively impacted Taiwan’s health industry and its spatial distribution.

## 8. Conclusions

The COVID-19 pandemic took place in 2020 and its aftermaths toward industries have led the academic world to investigate into the details of real situations and come up with effective solutions The purpose of this study is to provide the medical industry an experience of how health-related manufacturing industries in Taiwan were impacted during the period of COVID-19 from a spatial distribution perspective. This research fills the gap by looking into the relation between spatial concentration and survival performance of the health industry, a knowledge-intensive industry, under the pandemic situation.

Throughout our study, we have reached the following conclusions:

Firstly, based on the above analysis of industrial space survival, spatial distribution, and market trends of the HRMI, we found that for the number of industries and the performance of industrial space survival improved during the global outbreak of COVID-19 pandemic, although the progress in 2018–2019 was greater than that in 2019–2020. This indicates the overall growth of the HRMI in Taiwan before and after COVID and that the pandemic has not shocked the local health industry. From a viewpoint of life cycle theory, as the evolution stage an industry is in may impact its survival performance, especially during times of shock, we may consider the factor of life cycle stages somehow contributing to the survival of the evaluated industries.

Secondly, to look into the industrial distribution of the HRMI in Taiwan, we observe that its growth is concentrated predominantly in urban areas, specifically around metropolitans along the west coast. The reason behind the spatial distribution trend—also the reason for continuous growth of the HRMI—could be due to the nature of the specific industrial type (knowledge-intensive industry) also under the emergency circumstances of the global pandemic. We found the distribution of the HRMI in Taiwan generally matches the distribution of medical institutions and science parks. As health-related industries are often highly knowledge-intense, we conclude that the existence of neighbouring academic units and science parks may have played a significant role in supporting the HRMI, especially under the pandemic.

## Figures and Tables

**Table 1:** Descriptions and definitions of eight different categories of the HRMI.

Category	Description	Definition
2001	Manufacture of Raw Material Medicines	Industries engaged in the production of pharmaceutical raw materials for human or animal use, by synthesis, extraction, fermentation, tissue culture, etc.
2002	Manufacture of Drugs and Medicines	Processing of Western medicines for humans or animals, into certain doses and dosage forms.
2003	Manufacture of Medicinal Biological Products	Processing and modulation of medical biological products, such as biological drugs, vaccines, bacterins, serum, plasma extracts, etc.
2004	Manufacture of Chinese Medicines	Processing of traditional Chinese medicine for human or animal use, into certain doses or dosage forms.
2005	Manufacture of Medicinal Chemical Products	Manufacture of medical chemicals, such as in vitro diagnostic reagents, biochips, medical sutures, bandages, sterile gauze, dressings, etc.
2760	Manufacture of Irradiation and Electromedical Equipment	Industries engaged in the manufacture of radiation and electronic medical equipment, such as ultrasonic equipment, electronic hearing aids, and electronic medical endoscope equipment. Manufacturing of food sterilisation radiation equipment is also included in this category.
3321	Manufacture of Eyeglasses	Manufacturing of spectacles, such as corrective spectacles, sunglasses, contact lenses, diving glasses, safety goggles, etc. Spectacle frames and prosthetic eyes is also included in this category.
3329	Manufacture of Other Medical Instruments and Supplies	Manufacturing of non-electronic medical equipment and supplies other than those in category 3321, such as syringes, injection needles, catheters, artificial limbs, dentures, dental moulds, dental cement, dental appliances, and operating tables.

**Table 2:** Statistics of spatial death, survival and birth of different categories.

Category	Item	Number			Ratio			Difference		
		2018 (A)	2019 (B)	2020 (C)	2018	2019 (D)	2020 (E)	Number (B − A)	Number (C − B)	Ratio (E − D)
2001	Spatial death	--	9	4	--	18.00%	8.70%	--	−5	−9.30%
	Spatial survival	--	32	37	--	64.00%	80.43%	--	+5	16.43%
	Spatial birth	--	9	5	--	18.00%	10.87%	--	−4	−7.13%
	Total	41	50	46	100.00%	100.00%	100.00%	+9	−4	0.00%
2002	Spatial death	--	13	9	--	6.53%	4.48%	--	−4	−2.06%
	Spatial survival	--	165	177	--	82.91%	88.06%	--	+12	5.15%
	Spatial birth	--	21	15	--	10.55%	7.46%	--	−6	−3.09%
	Total	178	199	201	100.00%	100.00%	100.00%	+21	+2	0.00%
2003	Spatial death	--	9	1	--	18.37%	2.38%	--	−8	−15.99%
	Spatial survival	--	28	39	--	57.14%	92.86%	--	+11	35.71%
	Spatial birth	--	12	2	--	24.49%	4.76%	--	−10	−19.73%
	Total	37	49	42	100.00%	100.00%	100.00%	+12	−7	0.00%
2004	Spatial death	--	11	4	--	10.00%	3.74%	--	−7	−6.26%
	Spatial survival	--	89	95	--	80.91%	88.79%	--	+6	7.88%
	Spatial birth	--	10	8	--	9.09%	7.48%	--	−2	−1.61%
	Total	100	110	107	100.00%	100.00%	100.00%	+10	−3	0.00%
2005	Spatial death	--	7	3	--	9.46%	4.00%	--	−4	−5.46%
	Spatial survival	--	56	64	--	75.68%	85.33%	--	+8	9.66%
	Spatial birth	--	11	8	--	14.86%	10.67%	--	−3	−4.20%
	Total	63	74	75	100.00%	100.00%	100.00%	+11	+1	0.00%
2760	Spatial death	--	14	2	--	14.00%	2.11%	--	−12	−11.89%
	Spatial survival	--	68	84	--	68.00%	88.42%	--	+16	20.42%
	Spatial birth	--	18	9	--	18.00%	9.47%	--	−9	−8.53%
	Total	82	100	95	100.00%	100.00%	100.00%	+18	−5	0.00%
3321	Spatial death	--	24	9	--	8.16%	3.23%	--	−15	−4.94%
	Spatial survival	--	245	261	--	83.33%	93.55%	--	16	10.22%
	Spatial birth	--	25	9	--	8.50%	3.23%	--	−16	−5.28%
	Total	269	294	279	100.00%	100.00%	100.00%	+25	−15	0.00%
3329	Spatial death	--	83	49	--	11.05%	5.95%	--	−34	−5.11%
	Spatial survival	--	556	619	--	74.03%	75.12%	--	63	1.09%
	Spatial birth	--	112	156	--	14.91%	18.93%	--	44	4.02%
	Total	639	751	824	100.00%	100.00%	100.00%	+112	+73	0.00%

Note: The performance of spatial survival needs to be compared with the previous year, so it is not possible to further define the spatial death, spatial survival and spatial birth of 2018.

**Table 3:** Z-score of ANN analysis for spatial distribution of the HRMI.

Category	Z-Score			Concentration Growth Rate		
	2018 (A)	2019 (B)	2020 (C)	Rate a (B − A)/A	Rate b (C − B)/B	Rate c (C − A)/A
2001	−3.644782	−7.256016	−6.2034990	99.08%	−14.51%	70.20%
2002	−17.087448	−18.448492	−18.2726500	7.97%	−0.95%	6.94%
2003	−3.183437	−6.671437	−4.0516870	109.57%	−39.27%	27.27%
2004	−12.047356	−12.518739	−11.1341070	3.91%	−11.06%	−7.58%
2005	−9.445751	−9.429565	−9.6330170	−0.17%	2.16%	1.98%
2760	−9.452365	−10.256035	−10.473604	8.50%	2.12%	10.80%
3321	−20.668366	−20.271142	−20.268541	−1.92%	−0.01%	−1.93%
3329	−32.940749	−34.528372	−40.894399	4.82%	18.44%	24.15%

**Table 4:** LISA Analysis─COType Quantity Table.

Category	Type of Spatial Units	2018	2019	2020
2001	HH	8	8	8
	HL	29	30	31
2002	HH	42	40	40
	HL	94	95	100
2003	HH	5	5	5
	HL	28	29	29
2004	HH	29	27	25
	HL	59	59	61
2005	HH	12	14	16
	HL	40	42	42
2760	HH	24	26	31
	HL	35	32	33
3321	HH	85	75	73
	HL	65	70	70
3329	HH	213	197	221
	HL	157	154	159

**Table 5:** Industrial Spatial Operation Preference (Pr).

Category	2018	2019	2020
2001	0.28	0.27	0.26
2002	0.45	0.42	0.40
2003	0.18	0.17	0.17
2004	0.49	0.46	0.41
2005	0.30	0.33	0.38
2760	0.69	0.81	0.94
3321	1.31	1.07	1.04
3329	1.36	1.28	1.39

## Data Availability

The data presented in this study are available on request from the author.
